# Shared Decision Making: Aids and Promotion in Clinical Practice Guidelines for Type 2 Diabetes Treatment

**DOI:** 10.1111/jep.70457

**Published:** 2026-05-15

**Authors:** Yves Marie Vincent, Merwan Kasbi, Reda Saouli, Blanchard Clara, Rémy Boussageon

**Affiliations:** ^1^ Department of General Medicine Bordeaux University Bordeaux France; ^2^ Claude Bernard Lyon 1 University Lyon France; ^3^ Department of General Medicine Poitiers University Poitiers France; ^4^ UMR 5558, CNRS, LBBE, EMET Claude Bernard Lyon 1 University Lyon France

## Abstract

**Introduction:**

Type 2 diabetes (T2D) is a major global health issue due to its rising prevalence and significant burden in terms of morbidity, mortality, and economic impact. Although the availability of Clinical Practice Guidelines (CPGs) aimed at optimising T2D management, the complexity of therapeutic options and the necessity of tailoring treatment to individual patient preferences pose ongoing challenges. Shared Decision Making (SDM) and the high‐quality decision aids have been shown to enhance patient understanding and engagement in medical decisions. This study aimed to assess the presence of decision aids and the promotion of SDM within international and national CPGs for T2D treatment.

**Methods:**

Fifty‐two CPGs identified in a prior systematic review were re‐evaluated. Following the application of exclusion criteria and review of updates, 33 CPGs from 20 countries were included. The analysis focused on identifying high‐quality decision aids—defined as those providing absolute risk estimates for therapeutic options—and on detecting explicit support for SDM using predefined key terms.

**Results:**

No CPGs included decision aids that met the criteria for high quality. However, 73% acknowledged the importance of patient values and preferences, incorporating at least one reference to SDM. Despite this, no tools were provided to facilitate the practical implementation of SDM. This finding underscores a persistent gap between the theoretical recognition of SDM and its operational integration into CPGs.

**Discussion:**

These findings suggest that although the role of SDM is theoretically embraced, its practical application remains insufficient. Efforts from major guideline‐developing organisations to improve methodological inclusion of patient‐centred elements are encouraging, yet much progress is needed. Future guideline development must prioritise the integration of rigorously designed decision aids to truly support informed, value‐based medical decisions in T2D care.

## 1 Introduction

### 1.1 Diabetes and CPGs

Diabetes represents a global public health challenge. The global prevalence of diabetes in 2019 was 9.3%, corresponding to approximately 463 million people [1]. This figure is expected to rise to 10.2% (578 million) by 2030% and 10.9% (700 million) by 2045 [1]. Complications associated with diabetes, such as cardiovascular disease, kidney failure, retinopathy and neuropathy, contribute significantly to patient morbidity and mortality [2]. In a 2013 study of 187 countries, it was even found to be the 2nd most important factor in reducing life expectancy worldwide [3]. The economic impact is also significant, with an estimated cost of around $412.9 billion per year in the United States in 2022 [4] and a steady increase of 35% over 10 years.

Type 2 diabetes (T2D) is more specifically involved, with a higher prevalence than type 1 diabetes, with a rate of 0.5% of type 1 diabetes in the United States compared with 8.5% of T2D [5]. T2D has also been associated with an increase in morbidity and mortality over the last 30 years, whereas that of type 1 diabetes has been improving [6]. The challenge is therefore particularly acute in the case of T2D.

In view of these challenges, good clinical practice guidelines (GCP) play an essential role in providing healthcare professionals with evidence‐based guidelines for optimising the management of T2D. These recommendations cover various aspects, including glycaemic targets, therapeutic choices, management of co‐morbidities and therapeutic patient education. They are usually published by the leading learned societies in the field or by government institutions [7, 8, 9].

### 1.2 The Place of Shared Medical Decision‐Making

Despite the existence of these CPGs, the management of T2D remains complex due to the diversity of therapeutic options and the need to adapt treatments to the preferences, values and circumstances of each patient. Shared decision making (SDM), particularly through the use of decision support tools, has been identified as a means to improve both ethical practice and patients' understanding of therapeutic options and associated risks (Table 1) [10, 11].

**Table 1:** Various ways of presenting identical data on the effects of intensified vs conventional therapy on ‘any diabetes‐related endpoint’ (from I. Mühlhauser and M. Bergert, «evidence‐based patient information in diabetes» [10]).

	Intensified therapy	Conventional therapy
	100 patients over 10 years	100 patients over 10 years
	Median HbA_1c_ 7%	Median HbA_1c_ 7.9%
Patients with at least one endpoint		
No. of patients (%)	41 (41%)	46 (46%)
Patients without any endpoint		
No. of patients (%)	59 (59%)	54 (54%)
Differences		
Benefit of intensified versus conventional therapy		
Decrease in patients with endpoint		
No. of patients	5	
Absolute risk reduction	5%	
Relative risk reduction	11%	
Increase in patients without endpoint		
No. of patients	5	
Absolute increase	5%	
Relative increase	9%	
Lack of benefit of intensified versus conventional therapy		
Patients with an endpoint despite intensified therapy		
No. of patients	41	
Absolute per cent	41%	
Relative per cent (41 of 46)	89%	
All patients who do not benefit		
No. of patients (%)	95 (95%)	

A number of studies have shown that the use of properly developed decision aids leads to a better understanding of probabilities [12] and risks [13]. To do this and to be considered a high‐quality decision aid, the tools must present the different probabilities associated with the various choices available to patients [12, 13]. The different choices must include different treatment options as well as no‐treatment options, while complying with the international reference recommendations for patient decision aids published by IPDAS [14].

There are many examples of tools of this type, particularly in the field of dyslipidemia and treatment with lipid‐lowering drugs for primary prevention [15, 16]. Several players are developing this type of tool for T2D. The tools can be developed for specific points, such as when metformin is not sufficient to control diabetes [17]. They can also be more comprehensive in terms of managing the disease as a whole and be adapted to the characteristics of the patients [18].

Decision‐support tools have been shown to significantly improve patients' knowledge and risk perception, while reducing decisional conflict and passivity when making choices. In the specific case of T2D, several meta‐analyses and systematic reviews have found similar impacts [19, 20].

On the other hand, overly rigorous monitoring of CPGs can lead to risks for patients, particularly polypathological patients: the description of specific cases has led several researchers to advocate the wider inclusion of decision‐support tools in recommendations relating to T2D [21].

Given the beneficial impact of decision support tools in the care of T2D, the aim of this study is to evaluate the presence of decision support tools and the promotion of shared medical decision‐making in the CPGs on the treatment of T2D.

## 2 Methods

### 2.1 Search Strategy

To determine which CPGs to analyse, we decided to select the most widely used CPGs in the West, choosing documents published by the leading learned societies in Europe and North America, as well as international organisations. We based our work on a preliminary study of best practice recommendations for T2D [22] to identify these CPG creators.

For European learned societies, we have selected transnational learned societies: the European Society of Cardiology (ESC), the European Association for the Study of Diabetes (EASD) and the Société Francophone du Diabète (SFD). For national learned societies in Europe, we have retained the CPGs of the reference learned societies in England with the National Institute for Health and Clinical Excellence (NICE), France with the Haute Autorité de Santé (HAS), the Scottish Intercollegiate Guidelines Network (SIGN), the Revue Médicale de Liège (RML) in Belgium, and the Swiss Society of Endocrinology and Diabetology (SSED‐SGED) in Switzerland.

For North American learned societies, we have chosen Diabetes Canada as the reference society for Canada, for the United States, we have selected three societies that are recognised worldwide: the American Diabetes Association (ADA), the American College of Physicians (ACP) and the Department of Veterans Affairs (VA/DOD).

Finally, for international organisations, we have included the World Health Organisation (WHO) and the International Diabetes Federation (IDF).

We chose to use the latest CPGs published by these bodies, regardless of their date of publication, selecting the latest available version.

### 2.2 Analysis

The analysis was carried out by two researchers trained in the use of qualitative methods. Each researcher carried out an assessment before pooling the results and comparing them. In the event of disagreement, a third researcher could be called upon, but this was not necessary.

The primary objective was to identify high‐quality decision aids in CPGs, defined as tools presenting numerical estimates of absolute risk modifications—either increases or decreases—associated with each therapeutic option, rather than relative risk.

The secondary objective was to identify CPGs promoting SDM, defined as containing at least one recommendation supporting the importance of SDM. To achieve this, the following keywords were searched: “decision aid, discuss, informed decision, participate, partner, patient‐centred, patient choice, patient contribution, patient goal, patient involvement, patient priorities, preference, values, shared decision, share.” The presence of just one of these terms from this list, compiled based on MESH terms, was sufficient to validate this item.

## 3 Results

In total, we retrieved 14 CPGs [7, 9, 23, 24, 25, 26, 27, 28, 29, 30, 31, 32, 33, 34] published by the selected leading learned societies (Table 2). The oldest one dated from 2020, with all of the other 13 documents published between 2022 and 2026.

**Table 2:** List of recommendations analysed.

Organisations	Country	Publication date	Title
HAS	France	2024	Therapeutic strategy for patients living with T2D
SFD	French‐speaking countries	2023	Position statement by the Francophone Diabetes Society (SFD) on strategies for the use of antihyperglycaemic treatments in T2D
ESC	Europe	2019	*2019 ESC Guidelines on diabetes, pre‐diabetes and cardiovascular diseases were developed in collaboration with the EASD*
Revue Médicale de Liège	Belgium	2025	Update on the treatment of type 2 diabetes
SGED‐SSED	Suisse	2023	Recommendations of the Swiss Society of Endocrinology and Diabetology(SGED‐SSED) for the treatment of type 2 diabetes
SIGN	Scotland	2025	*Prevention and remission of type 2 diabetes*
NICE	United Kingdom	2022	*Type 2 diabetes in adults: management*
EASD and ADA	Europe and the United States	2022	*Standards of Care in Diabetes: Management of hyperglycaemia in type 2 diabetes, 2022. A consensus report by the American Diabetes Association (ADA) and the European Association for the Study of Diabetes (EASD)*
CDTA	Canada	2024	*Pharmacologic Glycemic Management of Type 2 Diabetes in Adults –2024 Update*
VA‐DOD	United‐states	2023	*VA/DoD clinical practice guideline for the management of type 2 diabetes mellitus*
ACP	United‐states	2024	*Newer Pharmacologic Treatments in Adults With Type 2 Diabetes: A Clinical Guideline From the American College of Physicians*
IDF	International	2025	*IDF Global Clinical Practice Recommendations for Managing Type 2 Diabetes*
WHO	International	2020	*Diagnosis and Management of Type 2 Diabetes*

Abbreviations: ADA, American Diabetes Association; ACP, American College of Physicians; ESC, European Society of Cardiology; EASD, the European Association for the Study of Diabetes (EASD); HAS, Haute Autorité de Santé; IDF, International Diabetes Federation; NICE, National Institute for Health and Clinical Excellence; SFD, Société Francophone du Diabète; SIGN, the Scottish Intercollegiate Guidelines Network; SSED‐SGED, Swiss Society of Endocrinology and Diabetology (SSED‐SGED); RML, Revue Médicale de Liège; VA/DOD, the Department of Veterans Affairs; WHO, World Health Organisation.

None of the 14 CPGs contained decision aids presenting numerical data on the benefits and risks of each therapeutic option with values of absolute risk influence.

Furthermore, all of the CPGs (14 out of 14) recognised the importance of taking patients' values or preferences into account in medical decisions.

## 4 Discussion

Decision aids should provide evidence‐based information on the benefits/risks of therapeutic options [35], and their presence is a quality criterion for the development of CPGs [36]. Therefore, their absence in the studied CPGs is concerning and does not push for informed choice among therapeutic options, especially considering that patients and clinicians tend to overestimate the benefits of therapeutic options while underestimating their risks [37, 38].

### 4.1 Quality and Criteria for Selecting Tools

Furthermore, some studies showed that the incorporation of decision aids into CPGs is frequently limited by their quality, with a non‐quantified or biased presentation of data with varying levels of evidence, which can lead to incorrect conclusions [39]. The presence of poor‐quality decision aids also raises questions about the methods used to develop CPGs: how do you choose which tool to present to patients and practitioners? This question is all the more important when good tools exist [19, 20].

The use of high‐quality tools benefits patients, particularly by enabling choices that are more in line with their values [40]. The use of these tools also improves satisfaction and reduces decision‐making conflicts among practitioners [41].

The recent update of the IPDAS's work on decision support tool design methods shows that, in parallel with implementation work in CPGs, decision support tool design criteria continue to improve and strengthen from methodological and scientific viewpoints [42].

### 4.2 Implementation: Which Method Should be Used to Develop It?

Despite the recognised importance of SDM in the studied CPGs, there is a significant and paradoxical gap in its implementation in practice. Similar results were found in the cardiovascular field, where 51% of CPGs support the importance of SDM, but only 3% contain decision aids [43]. Our study on diabetes, which is one of the most significant chronic diseases in terms of the number of patients affected and its impact on public health, confirms this gap and highlights the need to develop the presence of these tools in CPGs for chronic diseases.

A reference work on development methods develops the problems of internal and methodological tensions, which lead to giving priority to data from rigorous scientific data to determine rigid management by proposing a method giving more room for qualitative data and patient‐recorded outcomes [44]. Methodologists from major groups such as Veterans Affairs/Department of Defence (VA/DOD) in 2023 and National Institute of Health and Clinical Excellence (NICE) in 2024 are seeking to include these methodological elements in their CPG development methods. It would be relevant to assess the CPGs drawn up by learned societies that adapt their methodologies in this way.

These positive developments are in line with work in oncology, where the experts who draw up CPGs are aware of the need to include decision aids, but find it methodologically difficult to do so [45]. This encouraging data tends to suggest that the scientific community is aware of the problem, even if the approaches developed so far have clearly not yet been fruitful.

Thus, a concrete and significant step towards developing CPGs would be to apply the method already used by the VA/DOD: setting aside a place for qualitative data/data on patients' experiences in the data used for their design. In this regard, it should be noted that in 2022, the Spanish Ministry of Health published a guide entitled: Implementation of Clinical Practice Guidelines Recommendations in Shared Decision‐Making: Methodological Manual.

### 4.3 Strength and Weakness

These findings show the need to develop the integration of high‐quality into CPGs. These findings highlight the need to develop the integration of high‐quality decision aids into CPGs for the choice of treatments for T2D. Such tools exist [46] and provide clear information that leads to informed choices.

This study has several limitations. First, the selection of the CPGs studied is based on preliminary data, but it is not as robust as a literature review. Some CPGs published in other languages or those that are less accessible may have been omitted, limiting the representativeness of the sample. Finally, although two authors reviewed the CPGs, potential biases in data interpretation cannot be completely ruled out.

## 5 Conclusion

This study underscores the necessity of integrating high‐quality decision aids into CPGs for the treatment of T2D. Although SDM is recognised, the too‐rare presence of high‐quality decision aids limits patients' ability to make informed decisions. Such an evolution is essential to ensure optimal, patient‐centred care.

## Funding

The authors have nothing to report.

## Ethics Statement

This study meets the ethical requirements of French regulations.

## Conflicts of Interest

The authors declare no conflicts of interest.

## Data Availability

Data available on request from the authors. The data that support the findings of this study are available from the corresponding author upon reasonable request.
